# Mitigating bisphenol A-induced apoptosis in KGN cells: the therapeutic role of 1,25-dihydroxyvitamin D_3_ through upregulation of PGC-1α expression and inhibition of the mitochondrial cytochrome c pathway

**DOI:** 10.1007/s42000-024-00539-w

**Published:** 2024-02-29

**Authors:** Liting Tang, Ke Du, Kaiming Luo, Long Wang, Fei Hua

**Affiliations:** Department of Endocrinology and Metabolism, The First People’s Hospital of Changzhou, The Third Affiliated Hospital of Soochow University, Changzhou, 213003 China

**Keywords:** 1,25-dihydroxyvitamin D_3_, Bisphenol A, Apoptosis, KGN cells, Polycystic ovary syndrome

## Abstract

**Purpose:**

This study investigated the potential of 1,25-dihydroxyvitamin D_3_ (1,25(OH)_2_VD_3_) to mitigate bisphenol A (BPA)-induced apoptosis in human ovarian granulosa KGN cells with the aim of establishing a theoretical foundation for understanding of how vitamin D improved ovarian function in patients with polycystic ovary syndrome (PCOS).

**Methods:**

The impact of varying concentrations of BPA and 1,25(OH)_2_VD_3_ on KGN cell viability was elucidated. It was established that BPA-induced apoptosis in KGN cells. Subsequently, KGN cells underwent pretreatment with 1,25(OH)_2_VD_3_, followed by exposure to BPA. The apoptosis rate, reactive oxygen species (ROS) levels, and mitochondrial function of the cells were meticulously assessed, along with the expression levels of genes associated with apoptosis as well as antioxidant and mitochondrial biogenesis.

**Results:**

BPA induced a notable increase in apoptosis (*P* < 0.001) and oxidative stress (*P* < 0.001) in KGN cells, accompanied by a significant reduction in mitochondrial membrane potential (*P* < 0.001) and severe impairment of mitochondrial function. Following pretreatment of KGN cells with 1,25(OH)_2_VD_3_, there was a significant decrease in the apoptosis rate (*P* = 0.004), coupled with a reduction in ROS production (*P* = 0.002). Concomitantly, the upregulation of PGC-1α (*P* = 0.009) and SOD (*P* = 0.018) was observed, while mRNA expression of BAX (*P* = 0.011), Cyt c (*P* = 0.001), Apaf-1 (*P* = 0.012), caspase-9 (*P* < 0.001), and caspase-3 (*P* = 0.011) was downregulated. Notably, the mitigation of mitochondrial damage was evident through restored mitochondrial membrane potential (*P* < 0.001), as corroborated by electron microscope results.

**Conclusions:**

1,25(OH)_2_VD_3_ mitigated BPA-induced damage and apoptosis in KGN cells by upregulating the expression of PGC-1α and impeding the mitochondrial cytochrome c (Cyt c) apoptotic pathway. This study established a novel theoretical foundation for utilizing vitamin D in the treatment of PCOS patients.

**Supplementary Information:**

The online version contains supplementary material available at 10.1007/s42000-024-00539-w.

## Introduction

Polycystic ovary syndrome (PCOS) constitutes a multifaceted endocrine and metabolic anomaly prevalent among women of reproductive age, with an incidence ranging from 5 to 18%. Characterized by ovulatory dysfunction, disturbances in sex hormone metabolism, and polycystic ovarian changes, PCOS significantly impacts the reproductive health of affected individuals [1]. Despite its prevalence, the precise etiology of PCOS remains elusive, current research indicating potential associations with both environmental and genetic factors [2, 3].

Central to our investigation is 1,25-dihydroxyvitamin D_3_ (1,25(OH)_2_VD_3_), a steroid hormone recognized for its pivotal role in oocyte quality and reproductive function [4]. Animal studies have illuminated the impact of vitamin D deficiency, revealing its contribution to ovarian dysfunction in female rats [5]. In the clinical realm, vitamin D deficiency has been identified in certain PCOS patients, particularly those who are obese and hirsute [6]. Moreover, supplementation of vitamin D in PCOS patients has demonstrated positive effects, including improvements in blood glucose levels, insulin sensitivity, hyperlipidemia, and sex hormone levels. Of note, this supplementation has been associated with enhanced responses to ovulation induction, suggesting potential benefits for fertility and overall reproductive health [7–9, 10]. Nevertheless, the current understanding of the relationship between vitamin D and PCOS remains a subject of controversy, underscoring the need for further exploration.

Bisphenol A (BPA), a widespread endocrine-disrupting chemical (EDC) [3], has garnered attention due to elevated concentrations observed in patients with PCOS when compared to healthy individuals, and particularly correlating with hyperandrogenemia [11]. BPA exposure is implicated in potentially influencing human ovarian functions and disrupting normal folliculogenesis by activating autophagy in ovarian granulosa cells [12]. The identified abnormal function and increased apoptosis of ovarian granulosa cells in PCOS patients [13, 14] may be influenced by BPA exposure, further contributing to the pathogenesis of PCOS [15]. Consequently, we aimed to investigate whether vitamin D could mitigate the damage caused by BPA to ovarian granulosa cells.

Moreover, mitochondrial dysfunction in human ovarian granulosa cells has been suggested to compromise steroid hormone production and diminish oocyte quality, potentially impacting fertility [16]. Functional enrichment analysis has highlighted the fact that differentially expressed genes in ovarian granulosa cells in PCOS primarily involve oxidative phosphorylation and mitochondrial dysfunction [17]. However, the link between abnormal oocyte development in PCOS patients and mitochondrial dysfunction in ovarian granulosa cells remains unclear [18]. Previous studies indicate that BPA induces oxidative stress in ovarian granulosa cells [19], while vitamin D has been shown to enhance mitochondrial function [20]. Expanding on this groundwork, our study delved into the effects of BPA and 1,25(OH)_2_VD_3_ on mitochondrial function in human ovarian granulosa cells and sought to elucidate the underlying mechanisms.

In this investigation, we employed KGN cells known for their stability and physiological properties mirroring those of normal human ovarian granulosa cells. By inducing apoptosis in KGN cells through BPA exposure, we aimed to simulate the abnormal increase in apoptosis observed in ovarian granulosa cells of PCOS patients. Our hypothesis posited that vitamin D could ameliorate BPA-induced apoptosis and mitigate mitochondrial dysfunction in KGN cells.

## Materials and methods

### Cell culture and treatment

KGN cells (YS549C, Yaji Biology, China) were cultured in Dulbecco’s Modified Eagle Medium (DMEM)/F12 (BasalMedia, L310KJ, China) supplemented with 10% fetal bovine serum (FBS, BI, 04-001-1ACS, Israel) and 1% penicillin/streptomycin (Gibco, 15140-122, USA). Cells were maintained at 37 °C in a humidified atmosphere containing 5% CO_2_. After 24 h of seeding, the VD + BPA cell group underwent pretreatment with 1 µM 1,25(OH)_2_VD_3_ (Sigma, D1503, USA) dissolved in absolute dimethyl sulfoxide (DMSO, 0.1%) for 24 h. Subsequently, the medium was replaced, and the cells were treated with 100 µM BPA (Sigma, 239,658, USA) dissolved in absolute DMSO (0.1%) for an additional 24 h. The BPA cell group received the same concentration of DMSO for 24 h, followed by 24-h BPA treatment. The VD cell group was treated with the same concentration of 1,25(OH)_2_VD_3_ for 24 h and then exposed to DMSO for 24 h. The control group (CON) received the same concentration of DMSO each time the medium was changed.

### Cell viability assay (CCK-8)

Cell viability was assessed using the cell counting kit-8 (CCK-8, Beyotime, China) at 24, 48, and 72 h. Cells were seeded in 96-well plates at a density of 3000 cells per well and exposed to various compound concentrations for the specified durations. Briefly, 10 µL of working reagent was added to each well and the plates were incubated for 3 h at 37 °C. Absorbance was measured at 450 nm using a BioTek Elx 800 (BioTek, USA) plate reader.

### Flow cytometry analysis of cell apoptosis

Apoptosis was assessed using an Annexin V-FITC/PI kit (Multi Sciences, AP101, China). KGN cells from each group were seeded in 6-well plates at a density of 1 × 10^5^ cells per well. After treatment, the culture medium was discarded and the cells were washed with phosphate-buffered saline (PBS) before digestion with 0.25% trypsin-EDTA (Gibco, USA). Digestion was terminated in a medium containing 10% FBS, followed by centrifugation (1000 rpm, 5 min). A 1× working solution of 5× Binding Buffer was prepared and the cells were suspended in 500 µL of this working solution. Annexin V-FITC (5 µL) and PI (10 µL) were added to each tube, mixed, and incubated for 5 min at room temperature. Flow cytometric analysis was conducted using a BD Accuri™ C6 (Becton Dickinson, USA) cytometer.

### Reactive oxygen species (ROS) assay

Intracellular ROS levels were assessed using 2′,7′-dichlorofluorescein diacetate (Beyotime, China). KGN cells were seeded in 6-well plates at a density of 1 × 10^5^ cells per well. Following treatment with different compounds, 1 mL of 20 µM H_2_DCF-DA was added to each well and the plates were incubated at 37 °C for 30 min in the dark. Subsequently, the incubated cells were washed three times with a serum-free medium. A Hoechst stain working solution (Beyotime, China) was added to the cells and, after 5-min incubation at 37 °C, the cells were washed three times with serum-free medium. Images were captured using an IX71 microscope (Olympus, USA) in three randomly selected fields.

### Mitochondrial membrane potential detection

Mitochondrial membrane potential was measured using an enhanced mitochondrial membrane potential detection (JC-1) kit (Beyotime, C2003S, China). KGN cells were seeded in 6-well plates at a density of 1 × 10^5^ cells per well. Following the treatment with different compounds, the medium was discarded and the cells were washed twice with PBS. Subsequently, 300 µL of 0.25% trypsin-EDTA was added for cell digestion and the digestion was terminated in a medium containing 10% FBS. After centrifugation (1000 rpm, 5 min), the supernatant was discarded and 0.5 mL of medium was added. JC-1 (200×) was diluted with 1 mL of JC-1 staining buffer per 5 µL of JC-1 (200×). JC-1 dyeing solution (0.5 mL) was added to each tube and the tubes were incubated at 37 °C for 20 min. Following incubation, the tubes were centrifuged at 600 g for 4 min at 4 °C. The cells were washed twice with JC-1 staining buffer and resuspended in 1 mL of JC-1 staining buffer for flow cytometric analysis.

### Morphology assessment of cell mitochondria via electron microscopy

KGN cells (8 × 10^5^/well) were seeded in dishes and, after discarding the medium, the cells were washed twice with PBS. 1.5 mL of 2.5% glutaraldehyde was added to each dish and the cells were fixed at room temperature in the dark for 2 h. Gently removing the cells using cell scrapers followed by centrifugation at 2000 rpm for 5 min the supernatant was discarded and 1.5 mL of 2.5% glutaraldehyde was added to each tube. The cells were resuspended in the fixative via gentle pipetting and incubated at room temperature in the dark for 2 h. After incubation, the cells were stored at 4 °C or transported in 4 °C ice packs for further testing.

### RNA extraction and quantitative real-time polymerase chain reaction (qRT–PCR)

Total RNA was extracted from cells using TRIzol™ Reagent (Invitrogen, USA). The RNA concentration was determined spectrophotometrically with a Nanodrop One UV–vis spectrophotometer (ThermoFisher, USA). Subsequently, cDNA was synthesized using a PrimeScript™ RT Reagent Kit (Takara, Japan). qRT–PCR was conducted to assess the mRNA levels of BAX (Bcl-2-associated X protein), Bcl-2 (B-cell lymphoma-2), caspase-3 (cysteine aspartate protease 3), caspase-9 (cysteine aspartate protease 9), Cyt c (cytochrome c), Apaf-1 (apoptotic protease activating factor-1), and SOD (superoxide dismutase) using SYBR Green PCR Master Mix (Takara, Japan) on an ABI 7500 RT–PCR system (ThermoFisher, USA). GAPDH served as the endogenous control and the relative expression of the target genes was calculated employing the 2^△△CT^ method. The primer sequences are listed in Table 1.

**Table 1:** Primer sequences for qRT-PCR

Gene	Primer sequence
BAX	F: 5’ CCCGAGAGGTCTTTTTCCGAG 3’ R: 5’ CCAGCCCATGATGGTTCTGAT 3’
Bcl-2	F: 5’ GGTGGGGTCATGTGTGTGG 3’ R: 5’ CGGTTCAGGTACTCAGTCATCC 3’
caspase-9	F: 5’TAACAGGCAAGCAGCAAAGT3’ R: 5’ACCAAATCCTCCAGAACCAA3’
caspase-3	F: 5’AGAACTGGACTGTGGCATTG3’ R: 5’TAACCAGGTGCTGTGGAGTA3’
Apaf-1	F: 5’GCTAAAGACCGTCTCCGCA3’ R: 5’AGAGTCCCAAACATCATCCAAG3’
Cyt c	F: 5’CTTTGGGCGGAAGACAGGTC3’ R: 5’TTATTGGCGGCTGTGTAAGAG3’
SOD	F: 5’GGTGGGCCAAAGGATGAAGAG3’ R: 5’CCACAAGCCAAACGACTTCC3’
GAPDH	F: 5’TGGACCTGACCTGCCGTCTA3’ R: 5’CTGCTTCACCACCTTCTTGA3’

### Western blotting analysis

Cells were washed with cold PBS and lysed using RIPA lysis buffer (Beyotime, China). Total protein was extracted in the presence of protease inhibitors (ThermoFisher, USA) and protein concentration was determined using a BCA protein assay kit (Beyotime, China). Subsequently, equal amounts of proteins (20 µg per lane) were subjected to SDS–PAGE and then transferred onto a polyvinylidene difluoride membrane (ThermoFisher, USA). After blocking in 0.1% Tris-buffered saline with Tween-20 (TBST) containing 5% skim milk for 1 h at room temperature, the PVDF membrane was incubated overnight at 4 °C with primary antibodies against BAX (CST, 41,162 S, 1:1,000), Bcl-2 (CST, 4223 S, 1:1,000), caspase-3 (CST, 9662 S, 1:1,000), caspase-9 (CST, 9504 S, 1:1,000), Cyt c (CST, 11,940 S, 1:1,000), Apaf-1 (CST, 8969 S, 1:1,000), β-actin (CST, 4970 S, 1:1,000), and PGC-1α (peroxisome proliferator-activated receptor-coactivator-1α) (CST, 2178 S, 1:1,000). The membrane was rinsed three times with TBST, followed by incubation with a specific secondary antibody (CST, 7074P2, 1:1,000) for 1 h at room temperature. The protein levels were detected using an ECL detection solution (ThermoFisher Scientific, USA) and immunoreactive bands were visualized on a ChemiDoc Touch Imaging System (Bio-Rad, USA) and quantified using Image J software v1.49.

### Statistical analysis

Statistical analyses were conducted using GraphPad Prism 8.0 software. The results were expressed as means and standard error or standard deviation. The data were assessed using Student’s *t*-test for pairwise comparisons or ANOVA for multivariate analyses. A *P* value < 0.05 indicated significance (*P < 0.05; **P < 0.01; ***P < 0.001; and ****P < 0.0001).

## Results

### BPA decreases KGN cell viability in a time- and concentration-dependent manner

**Fig. 1:**
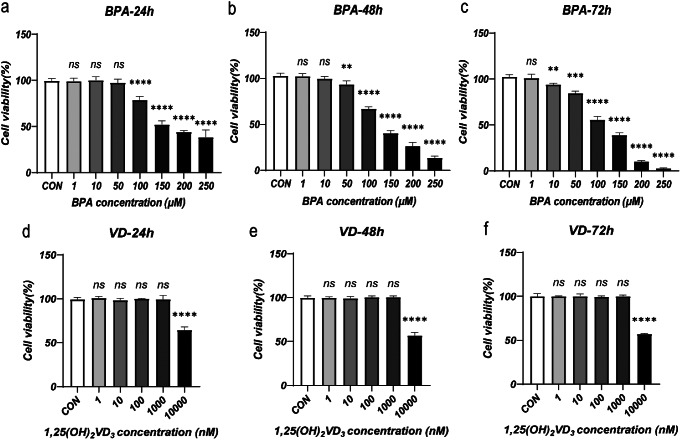
Effects of BPA and 1,25(OH)_2_VD_3_ on KGN cell viability with different concentrations and time intervals. The horizontal axis represents drug concentration, the vertical axis represents cell viability (%), and each group is compared with the control group (CON). Viability of KGN cells cultured with varying BPA (**a**, **b**, **c**) or 1,25(OH)_2_VD_3_ (**d**, **e**, **f**) concentrations for 24, 48, and 72 h. Data are shown as mean ± SD. ns *P* > 0.05, **P < 0.01, ***P < 0.001, and ****P < 0.0001

Cell viability was assessed in a medium containing varying BPA concentrations at 24 (Fig. 1a), 48 (Fig. 1b), and 72 h (Fig. 1c) post-treatment. Of interest, cell viability exhibited a significant reduction by 24 h at a BPA concentration of 100 µM (*P* < 0.001, Fig. 1a). As the BPA concentration and exposure time increased, the cell viability decreased. Conversely, treatment with different concentrations of 1,25(OH)_2_VD_3_ showed no significant impact on KGN cell viability at 24 (Fig. 1d), 48 (Fig. 1e), and 72 h (Fig. 1f) when the 1,25(OH)_2_VD_3_ concentration was less than or equal to 1 µM (1,000 nM) (*P* > 0.05, Fig. 1d, e, f). However, at a concentration of 10 µM (10,000 nM) 1,25(OH)_2_VD_3_, a significant inhibitory effect on cell viability was observed (*P* < 0.001, Fig. 1d, e, f). This finding indicated that 1,25(OH)_2_VD_3_ concentrations within 1 µM were more likely to exert protective effects on cells. In line with established research methods [20], we opted to pretreat KGN cells with 1 µM of 1,25(OH)_2_VD_3_ for 24 h based on the specific results of this experiment.

### BPA-induced changes in KGN cell activity result from induction of apoptosis

**Fig. 2:**
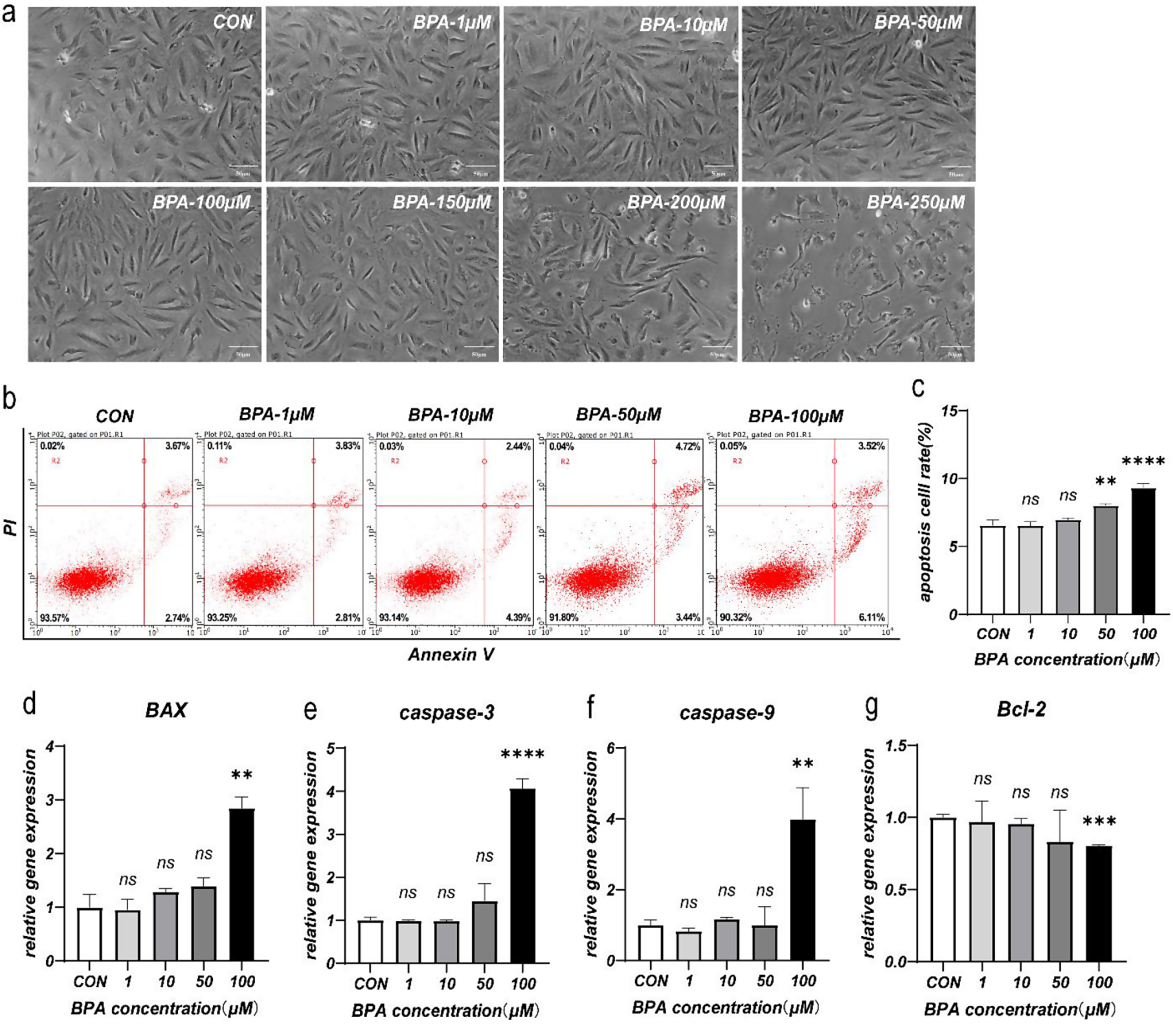
BPA induces apoptosis in KGN cells. KGN cells were treated with different BPA concentrations for 24 h. Each group is compared with the control group (CON). Morphological changes of KGN cells observed under light microscopy (**a**). Flow cytometry analysis of cell apoptosis in each group (**b**). Comparison of apoptosis rates among the groups (**c**); mRNA expression of apoptosis-related genes BAX (**d**), caspase-3 (**e**), caspase-9 (**f**), and Bcl-2 (**g**). Data are shown as mean ± SD. ns *P* > 0.05, **P < 0.01, ***P < 0.001, and ****P < 0.0001

Exposure of KGN cells to 100 µM BPA for 24 h induced noticeable morphological changes, including cell shrinkage and widened intercellular spaces (Fig. 2a). The apoptosis rate of KGN cells significantly increased (*P* < 0.001, Fig. 2b and c). Moreover, mRNA expression levels of BAX (*P* = 0.003, Fig. 2d), caspase-3 (*P* < 0.001, Fig. 2e), and caspase-9 (*P* = 0.005, Fig. 2f) genes were upregulated, while that of Bcl-2 (*P* < 0.001, Fig. 2g) markedly decreased. Therefore, the 24-h treatment of KGN cells with 100 µM BPA successfully established the BPA-induced apoptosis model.

### 1,25(OH)_2_VD_3_ alleviates BPA-induced KGN cell apoptosis

**Fig. 3:**
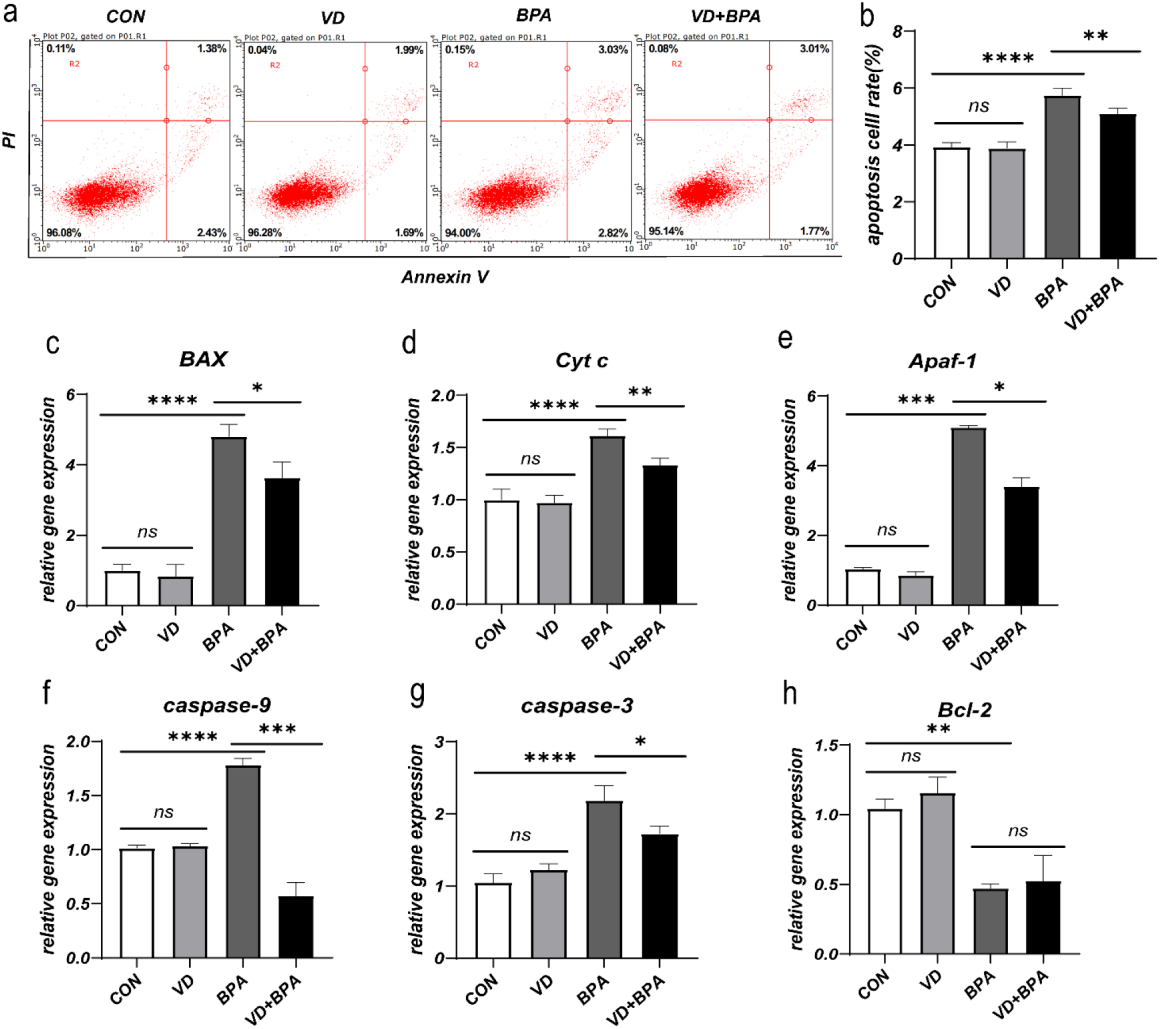
1,25(OH)_2_VD_3_ alleviates BPA-induced KGN cell apoptosis. Flow cytometry analysis of cell apoptosis in each group (**a**). Comparison of apoptosis rates among the groups (**b**); mRNA expression of apoptosis-related genes BAX (**c**), Cyt c (**d**), Apaf-1 (**e**), caspase-9 (**f**), caspase-3 (**g**), and Bcl-2 (**h**). Data are shown as mean ± SD. ns *P* > 0.05, *P < 0.05, **P < 0.01, ***P < 0.001, and ****P < 0.0001

**Fig. 4:**
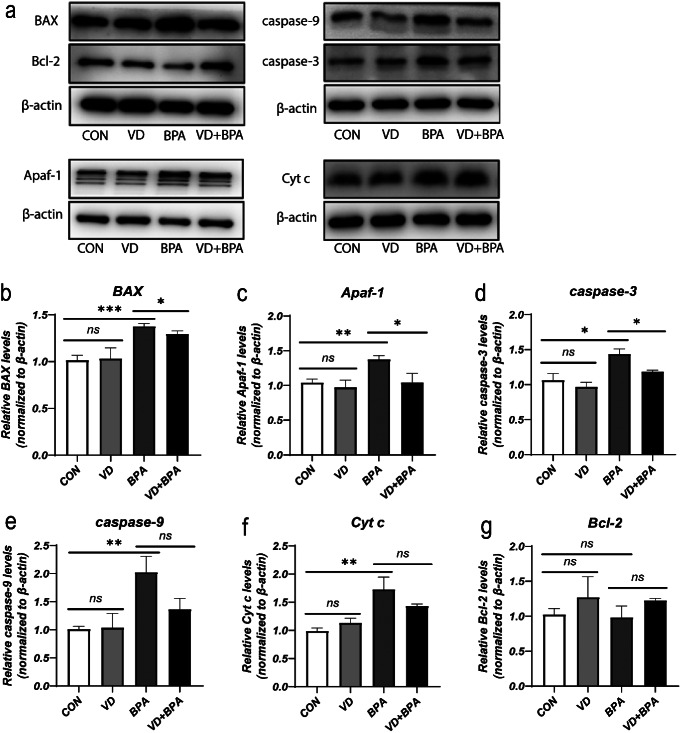
1,25(OH)_2_VD_3_ ameliorates BPA-induced protein expression alterations in mitochondrial apoptosis-related genes. Western blot plot (**a**). Quantitative analysis of protein expression for apoptotic genes BAX (**b**), Apaf-1 (**c**), caspase-3 (**d**), caspase-9 (**e**), and Cyt c (**f**), and the antiapoptotic gene Bcl-2 (**g**), with β-actin as a protein loading control. Data are shown as mean ± SD. ns *P* > 0.05, *P < 0.05, **P < 0.01, and ***P < 0.001

Following pretreatment with 1,25(OH)_2_VD_3_, there was a notable decrease in the apoptosis rate of KGN cells (*P* = 0.004, Fig. 3a and b). The mRNA expressions of apoptotic genes, including BAX (*P* = 0.011, Fig. 3c), Cyt c (*P* = 0.001, Fig. 3d), Apaf-1 (*P* = 0.012, Fig. 3e), caspase-9 (*P* < 0.001, Fig. 3f), and caspase-3 (*P* = 0.011, Fig. 3g) were downregulated. However, the difference in the mRNA expression level of antiapoptotic gene Bcl-2 was not statistically significant (*P* > 0.05, Fig. 3h). Furthermore, intervention with 1,25(OH)_2_VD_3_ significantly reduced the protein expression levels of BAX (*P* = 0.03, Fig. 4b), Apaf-1 (*P* = 0.046, Fig. 4c), and caspase-3 (*P* = 0.042, Fig. 4d). Conversely, the protein expression levels of caspase-9 (*P* > 0.05, Fig. 4e), Cyt c (*P* > 0.05, Fig. 4f), and Bcl-2 (*P* > 0.05, Fig. 4g) exhibited no significant differences in protein expression.

### 1,25(OH)_2_VD_3_ reduces BPA-induced oxidative stress in KGN cells

**Fig. 5:**
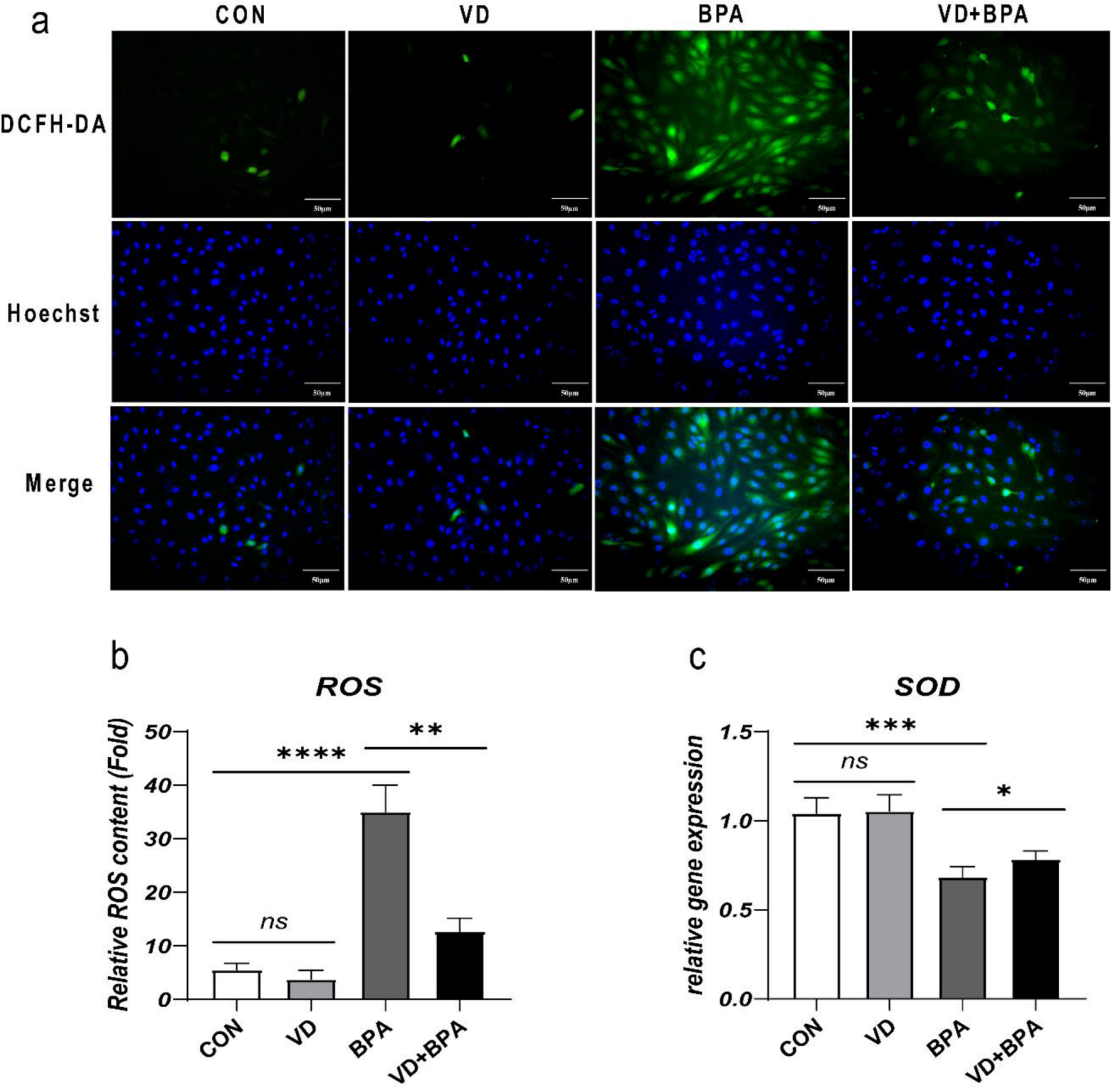
1,25(OH)_2_VD_3_ reduces BPA-induced oxidative stress in KGN cells. Diagram of ROS fluorescence (**a**); DCFH-DA green fluorescence shows ROS and Hoechst blue fluorescence shows the nuclei. Quantitative analysis of ROS fluorescence (**b**). qRT-PCR analysis of the antioxidant gene SOD (**c**). Data are shown as mean ± SD. ns *P* > 0.05, *P < 0.05, **P < 0.01, ***P < 0.001, and ****P < 0.0001

ROS production significantly increased in BPA-treated cells (*P* < 0.001, Fig. 5a and b) and decreased following 1,25(OH)_2_VD_3_ treatment (*P* = 0.002, Fig. 5a and b). Expression of SOD also significantly decreased in the BPA group (*P* < 0.001, Fig. 5c) in accordance with increased ROS production and significantly increased after 1,25(OH)_2_VD_3_ intervention (*P* = 0.018, Fig. 5c).

### 1,25(OH)_2_VD_3_ alleviates BPA-induced cellular mitochondrial damage

**Fig. 6:**
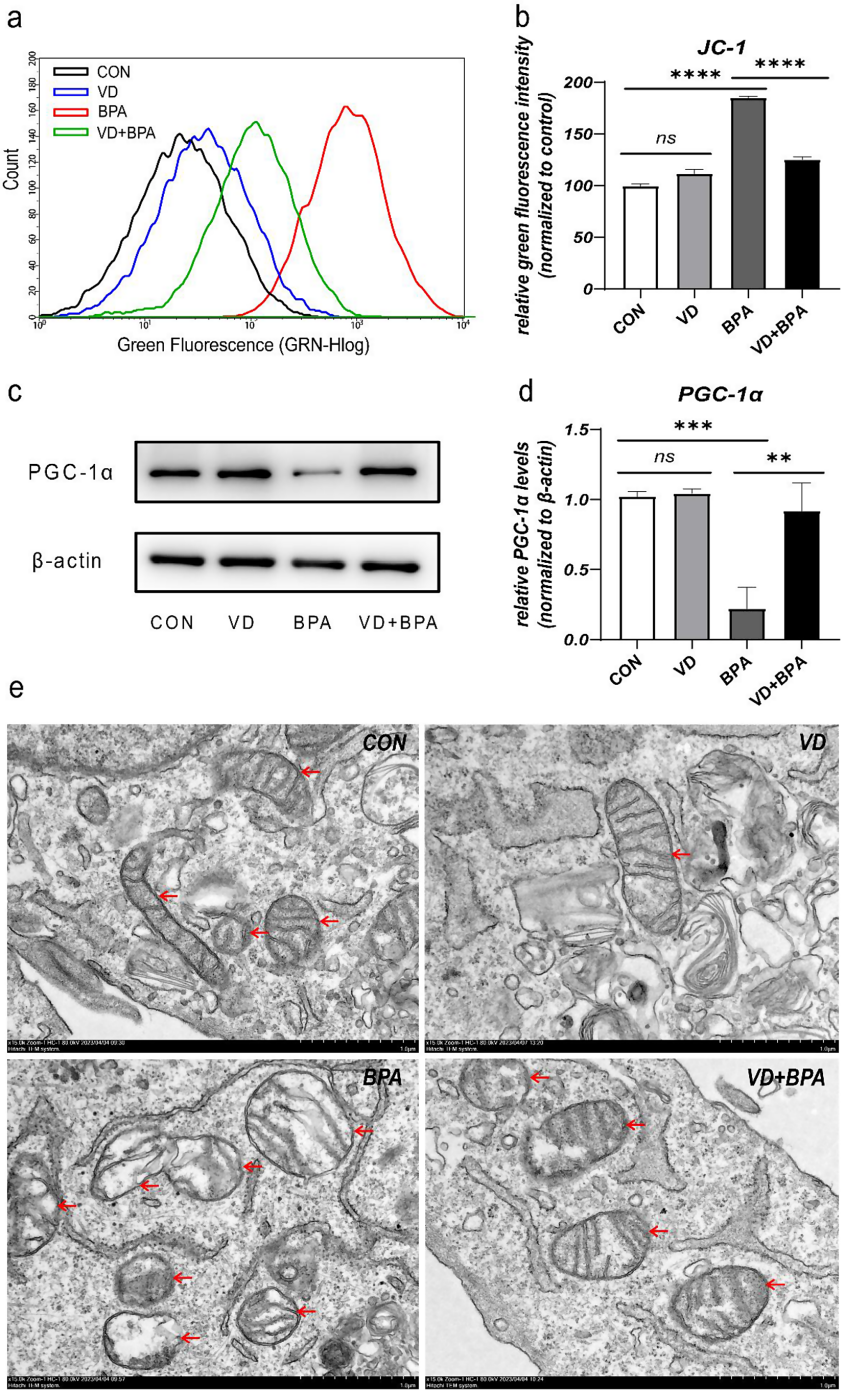
1,25(OH)_2_VD_3_ alleviates BPA-induced cellular mitochondrial damage. The changes of mitochondrial membrane potential in each group were detected by flow cytometry (**a**); JC-1 probe shows green fluorescence when the mitochondrial membrane potential decreases. Quantification analysis of JC-1 green fluorescence in each group (**b**). Western blotting analysis for PGC-1α (**c**), with β-actin as a protein loading control. Quantitative analysis of PGC-1α protein expression in each group (**d**). Mitochondrial electron micrographs: red arrows indicate mitochondria (**e**). Data are shown as mean ± SD. ns *P* > 0.05, **P < 0.01, ***P < 0.001, and ****P < 0.0001

The mitochondrial membrane potential of KGN cells significantly reduced after BPA treatment (*P* < 0.001, Fig. 6a and b) and the PGC-1α level significantly decreased (*P* < 0.001, Fig. 6c and d). The mitochondrial morphology displayed obvious abnormalities, with damaged mitochondrial membranes and disordered mitochondrial ridges (Fig. 6e). Post-1,25(OH)_2_VD_3_ intervention, mitochondrial function significantly improved, as evidenced by alleviation of the decline in mitochondrial membrane potential (*P* < 0.001, Fig. 6a and b), significant upregulation of PGC-1α expression (*P* = 0.009, Fig. 6c and d), and restoration of normal mitochondrial morphology, with clearly visible mitochondrial ridges in electron microscopy (Fig. 6e).

## Discussion

PCOS stands as a leading cause of anovulatory infertility among women of childbearing age. Despite its prevalence, the pathogenesis of PCOS remains elusive and effective therapeutic interventions are currently lacking. Significantly, recent research has implicated BPA in compromising reproductive function, suggesting its potential involvement in the development of PCOS. Concurrently, vitamin D deficiency has emerged as a potential factor in the pathogenesis of PCOS [21]. The intricate relationship between PCOS and vitamin D deficiency has long been unclear, with a recent study indicating that severe vitamin D deficiency is necessary before impacting ovarian function [22]. Clarifying a number of previous uncertainties, a randomized controlled clinical trial led by Al-Bayyari et al. has concluded that vitamin D_3_ administered at a treatment dose of 50,000 IU per week results in improved 25(OH)D levels. Additionally, it leads to reduced hirsutism scores and androgen levels in overweight women with PCOS [9].

Numerous studies have explored the impact of vitamin D on ovarian follicle production. For instance, 1,25(OH)_2_VD_3_ has been found to mitigate the effects of advanced glycation end products on anti-Mullerian hormone signaling [23] and alleviate DHEA-induced ferroptosis and oxidative stress in KGN cells [24]. Despite these findings, the specific mechanisms via which vitamin D operates in mitochondrial metabolism remain largely unknown. Moreover, limited research has as yet examined whether vitamin D can mitigate the detrimental effects of BPA on ovarian granulosa cells and the specific mechanisms underlying such mitigation. This knowledge gap underscores the need for further investigation into the intricate interplay between vitamin D, BPA, and ovarian function to pave the way for potential therapeutic avenues in PCOS management.

In this study, subsequent to BPA treatment of KGN cells, several key indicators demonstrated adverse effects, that is, while cell viability and mitochondrial membrane potential decreased, the apoptosis rate and ROS production increased. Additionally, the expressions of BAX, Apaf-1, Cyt c, caspase-9, and caspase-3 were upregulated, accompanied by downregulation of PGC-1α, SOD, and Bcl-2, along with severe mitochondrial damage. Following intervention with 1,25(OH)_2_VD_3_, a significant amelioration was observed. Apoptosis rate and ROS production were reduced and the decline in mitochondrial membrane potential was alleviated. The upregulation of apoptotic genes such as BAX, Apaf-1, Cyt c, caspase-9, and caspase-3 was reversed, while the expressions of PGC-1α and SOD were upregulated. Moreover, mitochondrial morphological changes and damage were markedly alleviated.

The effectiveness of 1,25(OH)_2_VD_3_ intervention in alleviating BPA-induced apoptosis and damage in KGN cells was attributed to the upregulation of PGC-1α expression and the inhibition of the mitochondrial Cyt c pathway. Notably, after 1,25(OH)_2_VD_3_ intervention, mRNA expression levels of caspase-9 and Cyt c were significantly downregulated, although protein expression levels did not show significant differences. This disparity might be attributed to rapid protein degradation, leading to high transcription levels and low protein levels. Alternatively, it could suggest an ongoing change in protein expression that had not yet reached a statistically significant level.

Studies have consistently demonstrated that BPA induces a substantial increase in oxidative stress in KGN cells [20, 25]. Beyond their central role in cellular energy metabolism, mitochondria play a crucial role in regulating apoptosis, with the mitochondrial pathway representing a key component of the endogenous apoptosis pathway [26]. PGC-1α, a pivotal gene for mitochondrial biogenesis, is intricately linked to oxidative stress, inflammation, and apoptosis [27]. A recent investigation has highlighted the fact that melatonin can mitigate BPA-induced apoptosis and damage in colon cells by upregulating PGC-1α expression and enhancing mitochondrial function [28]. Furthermore, an in vivo study by Safaei Z et al. has reported an increase in PGC-1α expression in granulosa cells after the administration of 1,25(OH)_2_VD_3_ in PCOS mice [29]. This aligns with our present study’s observation of the effect of 1,25(OH)_2_VD_3_ on BPA-induced apoptosis in KGN cells, suggesting that vitamin D may enhance mitochondrial function by upregulating PGC-1α expression.

While previous research has indicated that 1,25(OH)_2_VD_3_ rectifies BPA-disrupted mitochondrial function in rat granulosa cells [30], our findings revealed a novel aspect. Specifically, we confirmed, for the first time to our knowledge, that BPA treatment of KGN cells for 24 h can significantly downregulate PGC-1α expression, suggesting a potential association with apoptosis and oxidative stress. However, existing studies have yet to elucidate whether the regulation of PGC-1α expression is influenced by vitamin D receptors, while the precise molecular signaling pathway remains unknown. Consequently, further research is warranted to delve deeper into these aspects and provide a more comprehensive understanding.

Numerous studies have consistently highlighted the close association between mitochondrial dysfunction in ovarian granulosa cells and metabolic disorders in PCOS patients, underscoring the pivotal role of mitochondrial function in PCOS and infertility [31]. In a study involving PCOS mice, vitamin D_3_ is found to enhance mitochondrial biogenesis and membrane integrity in granulosa cells, potentially contributing to improved follicular development [20]. These findings align with our observations, where intervention with 1,25(OH)_2_VD_3_ significantly enhanced the mitochondrial function of KGN cells, effectively suppressing the expression of BPA-induced mitochondrial Cyt c signaling pathway-related apoptotic genes (BAX, Apaf-1, Cyt c, caspase-9, and caspase-3).

Bcl-2, by binding to BAX, inhibits BAX-induced decreases in mitochondrial transmembrane potential, thereby maintaining mitochondrial membrane integrity and counteracting apoptosis [32]. Interestingly, our study revealed that 1,25(OH)_2_VD_3_ had no significant effect on the expression of the antiapoptotic gene Bcl-2. In contrast, other studies have reported a significant upregulation of Bcl-2 expression in ovarian granulosa cells of PCOS mice following vitamin D_3_ treatment [29]. This disparity raises questions about whether the expression of the Bcl-2 gene may change after a more extended period of 1,25(OH)_2_VD_3_ intervention or if 1,25(OH)_2_VD_3_ exerts a more pronounced effect on proapoptotic genes than on antiapoptotic genes. Further exploration is needed to unravel the specific regulatory mechanisms and genomic changes underlying these observations.

Our research, while not without limitations, is currently undergoing refinement. Our focus entails inducing PCOS-related metabolic syndrome in rats through the administration of BPA. Subsequently, we plan to supplement vitamin D_3_ in vitro and analyze the metabolic changes in rats pre- and post-experimentation. Additionally, we aim to conduct genomic analysis on extracted ovarian granulosa cells.

The findings underscored the imperative of monitoring vitamin D levels in childbearing-age PCOS patients within clinical settings. Moreover, advocating against exposure to EDCs such as BPA is essential. Looking ahead, the prospect of quantifying BPA and vitamin D levels in PCOS patients holds promise for predicting their response to ovulation induction and prognosis. This personalized approach could revolutionize treatment strategies for individual patients.

Our study unequivocally established that BPA induced apoptosis and oxidative stress in KGN cells, leading to profound mitochondrial damage through the downregulation of PGC-1α gene expression. However, intervention with 1,25(OH)_2_VD_3_ markedly alleviated apoptosis, oxidative stress, and mitochondrial damage. This mitigation was achieved by upregulating PGC-1α and inhibiting the mitochondrial Cyt c signaling pathway. These compelling findings offer robust evidence supporting the beneficial impact of 1,25(OH)_2_VD_3_ on ovarian function in PCOS patients. The implications of our research are of paramount importance for advancing our understanding of PCOS pathogenesis and refining treatment modalities.

## Electronic supplementary material

Below is the link to the electronic supplementary material.

Supplementary Material 1

## Data Availability

Not applicable.
